# Small Molecules Targeted to a Non-Catalytic “RVxF” Binding Site of Protein Phosphatase-1 Inhibit HIV-1

**DOI:** 10.1371/journal.pone.0039481

**Published:** 2012-06-29

**Authors:** Tatiana Ammosova, Maxim Platonov, Venkat R. K. Yedavalli, Yuri Obukhov, Victor R. Gordeuk, Kuan-Teh Jeang, Dmytro Kovalskyy, Sergei Nekhai

**Affiliations:** 1 Center for Sickle Cell Disease, Howard University, Washington, D.C., United States of America; 2 RCMI Proteomics Core Facility, Howard University, Washington, D.C., United States of America; 3 Department of Medicine, Howard University, Washington, D.C., United States of America; 4 ChemBio Center, National Taras Shevchenko University, Kiev, Ukraine; 5 Institute of Molecular Biology and Genetics, National Academy of Sciences of Ukraine, Kiev, Ukraine; 6 Molecular Virology Section, Laboratory of Molecular Microbiology, National Institute of Allergy and Infectious Diseases, National Institutes of Health, Bethesda, Maryland, United States of America; 7 Sickle Cell Center, University of Illinois at Chicago, Chicago, Illinois, United States of America; George Mason University, United States of America

## Abstract

HIV-1 Tat protein recruits host cell factors including CDK9/cyclin T1 to HIV-1 TAR RNA and thereby induces HIV-1 transcription. An interaction with host Ser/Thr protein phosphatase-1 (PP1) is critical for this function of Tat. PP1 binds to a Tat sequence, Q^35^VCF^38^, which resembles the PP1-binding “RVxF” motif present on PP1-binding regulatory subunits. We showed that expression of PP1 binding peptide, a central domain of Nuclear Inhibitor of PP1, disrupted the interaction of HIV-1 Tat with PP1 and inhibited HIV-1 transcription and replication. Here, we report small molecule compounds that target the “RVxF”-binding cavity of PP1 to disrupt the interaction of PP1 with Tat and inhibit HIV-1 replication. Using the crystal structure of PP1, we virtually screened 300,000 compounds and identified 262 small molecules that were predicted to bind the “RVxF”-accommodating cavity of PP1. These compounds were then assayed for inhibition of HIV-1 transcription in CEM T cells. One of the compounds, 1H4, inhibited HIV-1 transcription and replication at non-cytotoxic concentrations. 1H4 prevented PP1-mediated dephosphorylation of a substrate peptide containing an RVxF sequence *in vitro*. 1H4 also disrupted the association of PP1 with Tat in cultured cells without having an effect on the interaction of PP1 with the cellular regulators, NIPP1 and PNUTS, or on the cellular proteome. Finally, 1H4 prevented the translocation of PP1 to the nucleus. Taken together, our study shows that HIV- inhibition can be achieved through using small molecules to target a non-catalytic site of PP1. This proof-of-principle study can serve as a starting point for the development of novel antiviral drugs that target the interface of HIV-1 viral proteins with their host partners.

## Introduction

The emergence of drug-resistant HIV-1 presents a challenge for the design of new therapeutics. Targeting host cell factors employed by HIV-1 for replication could be an approach to address HIV-1 drug resistance. Transcription of HIV-1 is dependent on the viral Tat protein, which binds a nascent trans-activation responsive (TAR) RNA [1] and recruits host cell transcription factors including CDK9/cyclin T1 to the viral LTR (reviewed in [2]). Phosphorylation of the RNAP II carboxyl-terminal domain (CTD) is functionally critical for HIV-1 transcription. Conversely, CTD-dephosphorylation mediated through protein phosphatase-1 (PP1) is similarly essential for HIV-1 transcription [3], [4]. Currently, it is thought that PP1 enters the viral transcriptional schema through direct recruitment by the ^35^QVCF^38^ motif of Tat [5], [6]. The PP1 holoenzyme consists of a constant catalytic subunit (PP1α, PP1β/δ or PP1γ) and a variable regulatory subunit that determines the localization, activity and substrate-specificity of the phosphatase [7]. Regulatory subunits bind to the catalytic subunit through one or more motifs, such as the well established RVxF motif and the recently identified SILK and MyPhoNE motifs [8]. Major PP1 regulators, such as NIPP1 (Nuclear Inhibitor of PP1) or PNUTS (Phosphatase Nuclear Targeting Subunit), bind PP1 with nanomolar affinity and modulate the dephosphorylation of a wide range of PP1 substrates [7]. Previously, we found that Tat binds PP1 in a similar manner to how NIPP1 binds PP1, except that the Tat-PP1 interaction is weaker and occurs with micromolar affinity [6].

Interestingly, a Q35R mutation in Tat conferred a higher affinity for PP1, although this mutation is inactivating for Tat-mediated transcription, potentially because a tighter association with PP1 prevents Tat-binding to CDK9/cyclin T1 [6]. Recent studies point to CDK9 as a potential target for PP1 dephosphorylation. The high molecular weight positive transcription elongation factor b (P-TEFb) contains CDK9/cyclin T1, 7SK RNA, HEXIM1 protein, and recently identified La-related protein (LARP7) and methylphosphatase capping enzyme (MePCE) [9], [10]. The high molecular weight P-TEFb complex plays an important role in the activation of HIV-1 transcription, as it serves as a source of CDK9/cyclin T1 for recruitment by HIV-1 Tat [11]. Dephosphorylation of CDK9 on Thr186 by protein phosphatase-1 (PP1) in stress-induced cells dissociates 7SK RNA and HEXIM1 and activates CDK9/cyclin T1 [12]. Our recent study showed that HIV-1 transcription and replication are inhibited in cells that stably express the central domain of NIPP1 (cdNIPP1) or when cdNIPP1 is expressed as part of HIV-1 pNL4-3 in place of *nef* [5]. The stable expression of cdNIPP1 disrupts the interaction of Tat with PP1 and also increases CDK9 phosphorylation of Thr186 and the association of CDK9 with 7SK RNA [5]. We also recently showed that PP1 dephosphorylates Ser175 of CDK9 and activates HIV-1 transcription [13] suggesting an additional activation mechanism for PP1 in HIV-1 transcription.

Thus, our previous studies showed that PP1 is important for the activation of HIV-1 transcription and that disruption of the HIV-1 Tat/PP1 interaction is inhibitory for HIV-1. These findings suggest the possibility of identifying small molecules that can act in a manner similar to the expression of cdNIPP1 to disrupt the interaction of Tat and PP1 and inhibit HIV-1 transcription. Here we report the identification of a small molecule, 1H4 that inhibits HIV-1 transcription and replication. This compound affected the binding of Tat’s RVxF motif to PP1 *in vitro* and the binding of Tat to PP1 in cultured cells but had no effect on the binding of PP1 to the major regulatory subunits, NIPP1 and PNUTS, or the expression of cellular proteins. We further analyzed the effect of 1H4 on the interaction of Tat with PP1α in cultured cells by comparing the distribution of PP1 between the cytoplasm and nucleus.

## Results

### Design of Small Molecule “RVxF” Mimetic Library

**Figure 1:**
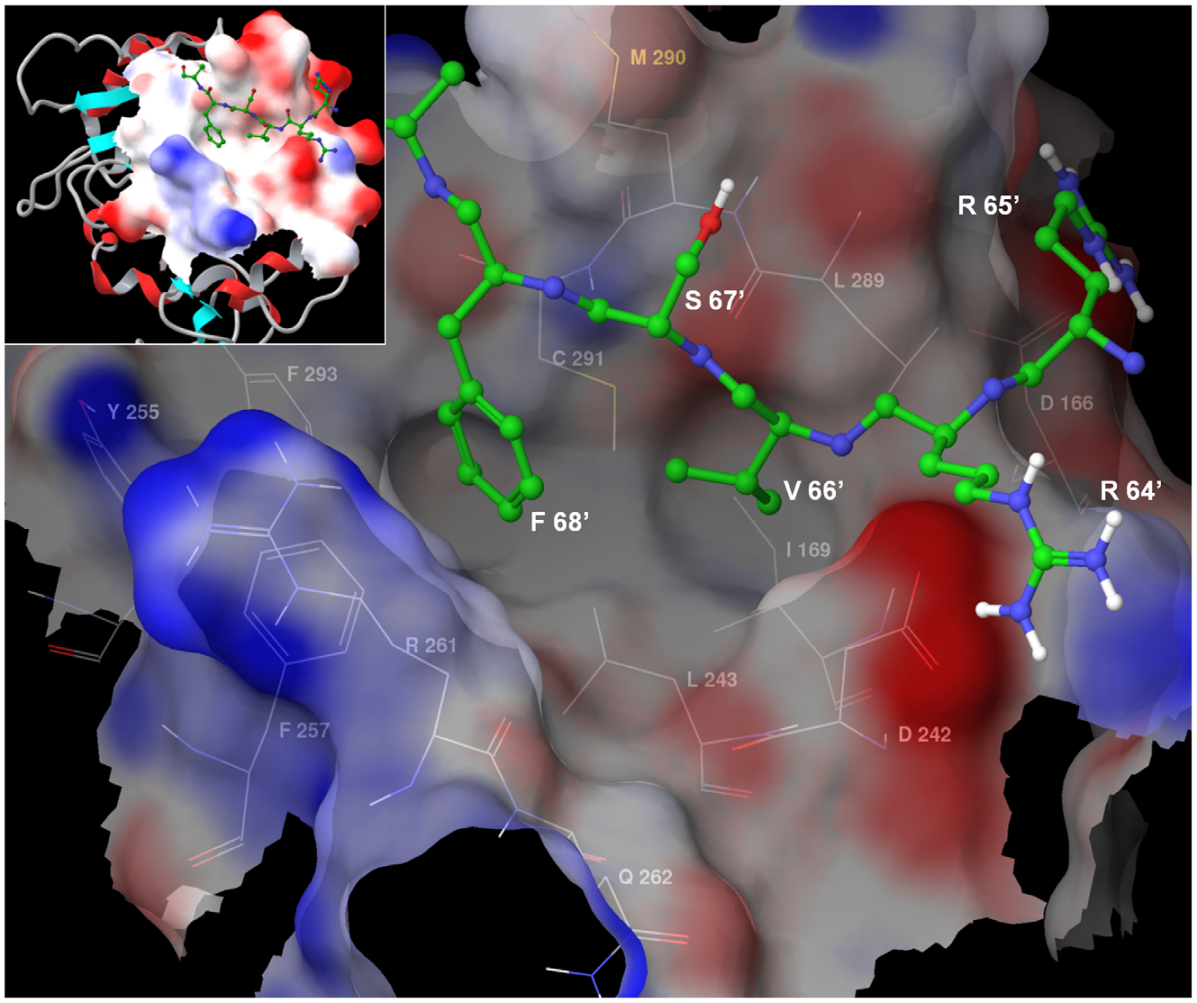
PP1 with RVxF peptide bound to its hydrophobic channel. Peptide RRVSFA derived from Gm protein, a regulatory subunit of PP1 involved in glycogen metabolism, shown in ball-and-stick representation and colored after the CPK scheme, with carbon atoms colored in green for clarity. Solvent accessible surface area around the RVxF binding site is shown in transparent, and colored according to electrostatic potential.

**Figure 2:**
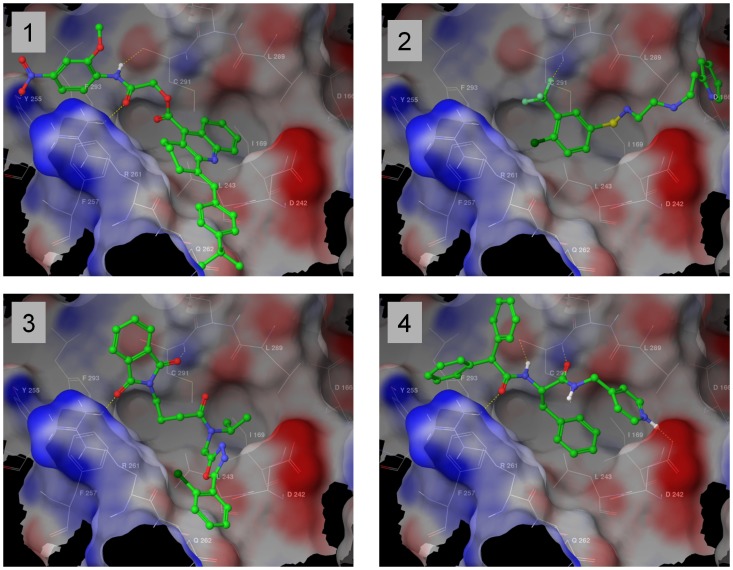
Four binding modes for PP1 inhibitors. Complexes with representative ligands, fulfilling each binding mode are shown. Representation scheme same as Figure 1. Hydrogen bonds are shown in yellow. Panel 1. Compounds are positioned toward Tyr255 were selected. Panel 2. Compounds reach Asp166. Panel 3. Compounds are span toward Gln262. Panel 4. Compounds form more than 4 hydrogen bonds with PP1.

We chose the complex of PP1γ with RRVSFA peptide [14] for docking experiments (X-ray coordinates courtesy of David Barford). The binding of the RVxF motif to PP1 in this complex is largely driven by van der Waals interactions of the valine and phenylalanine side chains [14]. In the ^64^RRVSFA^69^ peptide, Val66’ and Phe68’ side chains (Fig. 1) interact with a hydrophobic channel located on the opposite side of the catalytic center of PP1γ. The side chain of Phe68’ interacts with a site formed by the Leu243, Phe257, Cys291 and Phe293 residues of PP1γ while the Val66’ side chain binds to an adjacent site formed by Ile169, Leu243, Leu289 and Cys291 (Fig. 1). These two sites serve for tethering the RVxF motif to PP1γ. Hence, we envisioned that an ideal RVxF competing compound would occupy one or both of these sites. Arg65' makes a salt bridge interaction outside of the binding pocket, so we could not mimic this interaction with small molecules that occupy only the “RVXF”-accommodating cavity of PP1. Based on these considerations, the initial pharmacophore model was build to select ligands occupying the hydrophobic channel and forming at least two hydrogen bonds with PP1γ. Since the channel has a shallow depth preference was given to compounds that formed large contact surfaces. About 300,000 compounds from the Enamine (Kiev, Ukraine) stock collection were virtually screened for binding to PP1 (see description of the screening process in Materials and Methods). The resulting 1572 compounds were processed sequentially in two steps (described in Materials and Methods and outlined in Fig. S1). Rough filtering was employed to remove outliers and allowed to select compounds for further evaluation (step one, Fig. S1). Geometric filtering was used to select compounds that fell under one of four distinct binding modes (step two, Fig. S1). In the first mode, compounds filled a region near Tyr255 (Fig. 2, panel 1). In the second mode, compounds bound within 6.5 Å of Cβ of Asp166 (Fig. 2, panel 2). In the third mode, compounds bound within 4 Å of the amide oxygen of Gln262 (Fig. 2, panel 3). In the fourth mode, compounds were confined to the Val66’ and Phe68’ hydrophobic sub-sites and formed extensive hydrogen bonds with at least two of the following residues: Lys260, Arg261, Asp242, Val289, M290 and Cys291 (Fig. 2, panel 4). We obtained 262 compounds that collectively represented these four binding modes; these compounds were further evaluated biologically for inhibition of HIV-1 replication as described below.

### Identification of HIV-1 Inhibitory Compounds

We evaluated all 262 candidate compounds for inhibition of Tat-dependent HIV-1 transcription (Table S1) using a previously described [15] reporter assay. CEM-GFP cells containing LTR-GFP reporter were infected with an adenovirus expressing HIV-1 Tat and GFP fluorescence was detected on a microplate reader. Ad-Tat infected CEM-GFP cells were incubated with 25 µM of each compound for 48 hours to determine the inhibitory activity of the compound. Cytotoxicity was evaluated in the same plate by the addition of propidium iodide (PI) and measurement of red fluorescence.

**Figure 3:**
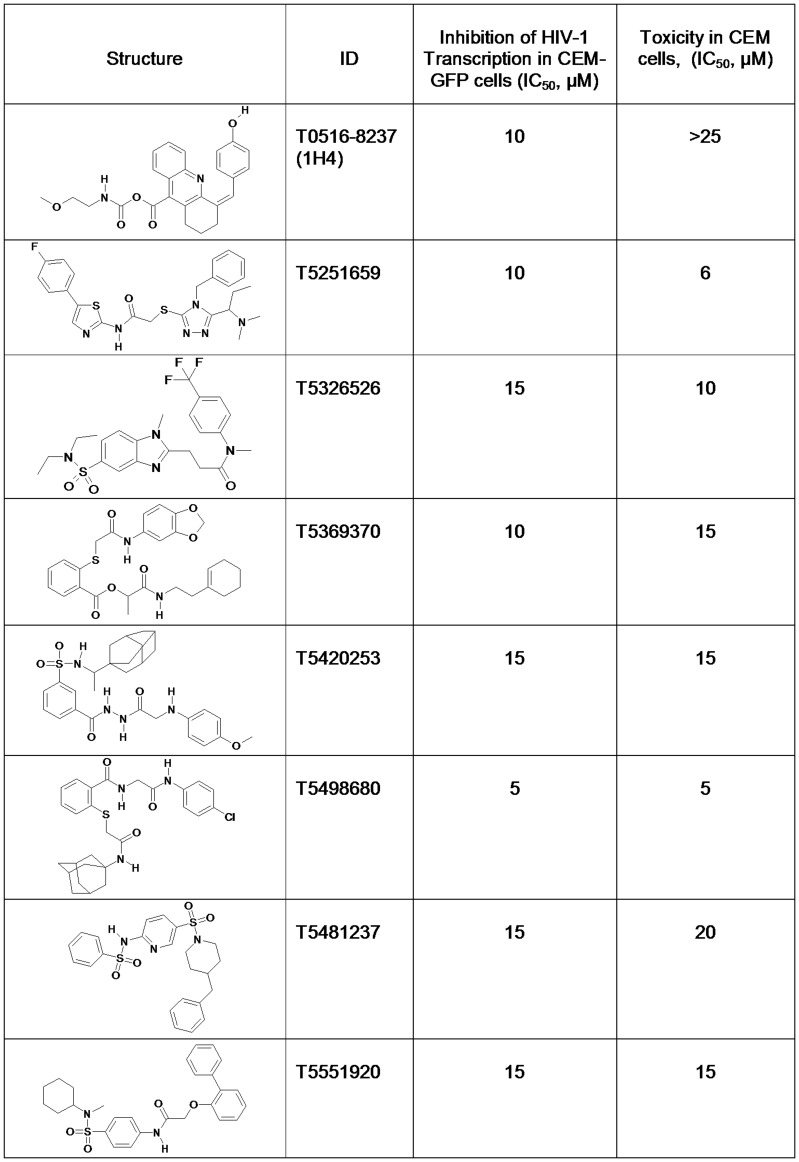
Selected compounds that inhibited HIV-1 transcription. CEM-GFP cells were infected with Adeno-Tat and then treated with the indicated compounds at concentrations between 3 µM to 45 µM for 24 h. GFP fluorescence was measured in live cells. The cells were supplemented with propidium iodide (PI), and its fluorescence was measured to determine the toxicity of the compounds.

Sixty compounds that inhibited HIV-1 transcription by at least 80% at 25 µM were found (Table S1, marked as gray). These 60 compounds were further analyzed to determine the IC_50_ for the inhibition of transcription. This dose-dependent analysis identified 17 compounds that inhibited HIV-1 transcription in CEM-GFP cells with IC_50_s below 25 µM and 8 compounds that inhibited HIV-1 transcription at IC_50_s below 15 µM (Fig. 3; see examples in Fig. S2A). Amongst the latter 8 compounds, 1H4 was not cytotoxic at the concentrations tested.

**Figure 4:**
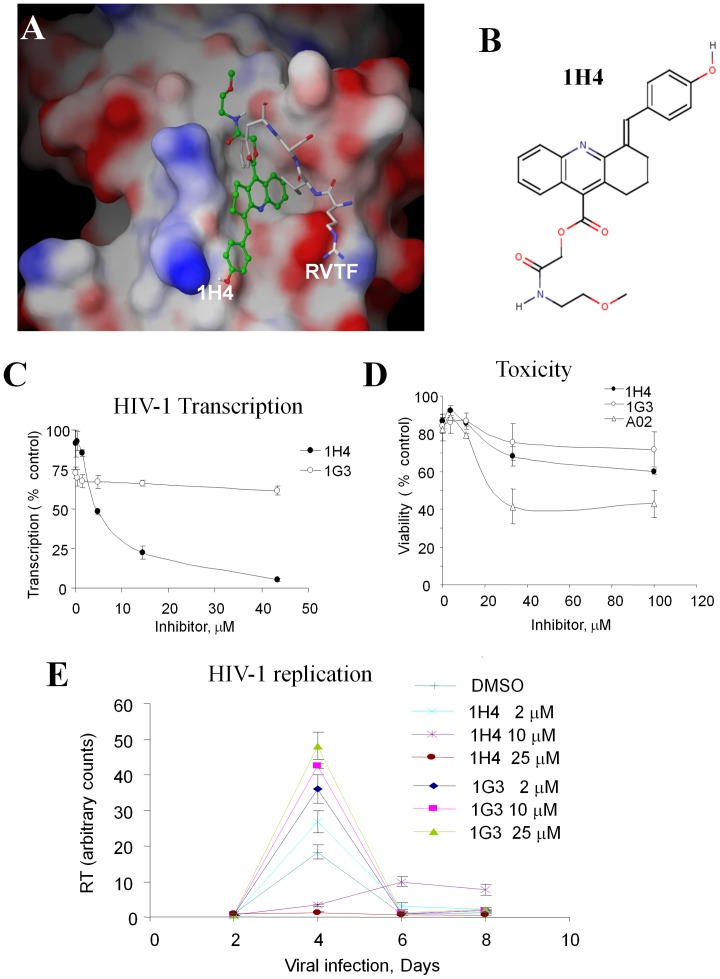
Inhibition of HIV-1 transcription and replication by 1H4. A. The model of 1H4 interaction with PP1. 1H4 occupies hydrophobic sites of Phe68’ and Val 66′ side chains, interacts with Gln262 and forms a network of hydrogen bonds with Arg261 and Cys291. B. Chemical structure of 1H4. C. Inhibition of HIV-1 transcription in 293T cells. 293T cells were transfected with HIV-1 LTR-LacZ, CEM-GFP and Tat expressing vectors and treated with the indicated concentrations of 1H4 and 1G3 compounds. At 24 hours after the transfection, the cells were lysed and analyzed for green fluorescence and for β-galactosidase activity. D. Toxicity in CEM cells. CEM cells were treated with the indicated concentrations of 1H4, 1G3 and toxic A02 compound for 24 hours. The viability was determined using trypan exclusion assay and automated cell counter (Nexcelcom). E. HIV-1 replication is inhibited by 1H4. MT4 cells were infected with recombinant pNL4-3 HIV-1 and treated with different concentrations of 1H4 or inactive control 1G3. The RT activities were determined over the course of 8 days.

The interaction of 1H4 with PP1 falls into the first binding mode (Fig. 4, panels A and B), although 1H4 did not quite reach Tyr255 as depicted in the prototype compound (Fig. 2, panel 1). We further evaluated 1H4 and five other compounds for inhibition of HIV-1 LTR-directed transcription in 293T cells transfected with a Tat-expressing vector and an HIV-1 LTR-*Lac Z* reporter [16]. In this assay, we found that 1H4 inhibited viral transcription with an IC_50_ of 5 µM (Fig. 4C); by contrast, the other tested compounds were not inhibitory at concentrations below 10 µM (not shown). Analysis of 1H4 in CEM cells by trypan blue exclusion assay showed that it was not cytotoxic (IC_50_>100 µM, Fig. 4D) in contrast to the toxic A02 compound that was used as a control (Fig. 4D). The effect of 1H4 was specific for the HIV-1 promoter as it did not inhibit transcription from a control CMV promoter (Fig. S2B). Taken together, we were able to identify a single compound, 1H4 that specifically inhibited HIV-1 transcription in cultured CEM and 293T cells.

### HIV-1 Replication is Inhibited by 1H4

Next, we determined the effect of 1H4 on productive HIV-1 replication. MT4 cells were infected with HIV-1 NL4-3, and the cells were treated with 2 µM, 10 µM or 25 µM concentrations of 1H4, and, as a control, an inactive compound 1G3. 1H4 inhibited HIV-1 replication beginning at the 10 µM concentration (Fig. 4E) while 1G3 did not inhibit HIV-1 replication (Fig. 4E). Thus 1H4 inhibited both HIV-1 transcription and replication.

### 1H4 Inhibited the Interaction of Tat with PP1 in vitro

**Figure 5:**
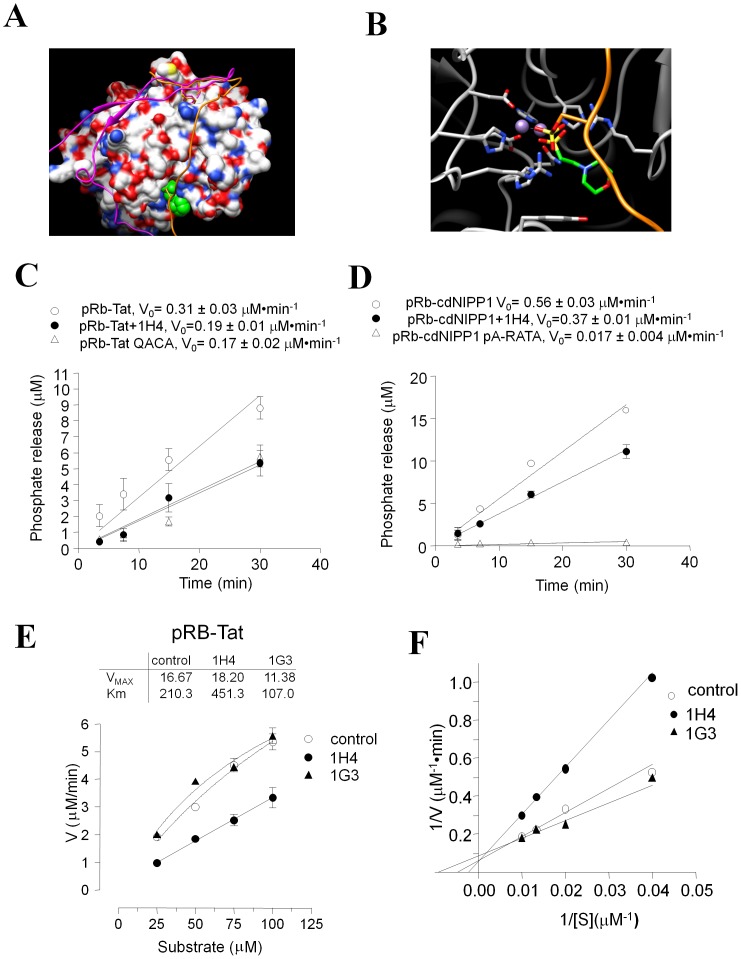
1H4 compound competes with RVxF motif. A. Superimposition of pRb-Tat peptide on the complex of PP1 with Spinophilin. PP1 surface is colored after the atom types. The spinophilin peptide is shown as magenta ribbon and the pRb-Tat peptide as orange ribbon. The Val25 and Phe27 residues of pRb-Tat and the Ile449 and Phe 451 residues of Spinophilin are shown as sticks. The 2-(N-morpholino)-ethanesulfonic acid bound in the active site of PP1 is shown in green spheres. The phosphorylated Ser6 residue of pRb-Tat peptide is shown as sticks. B. Phosphorylated Ser 6 residue binds to the active site of PP1. Comparative superimposition of pRb-Tat peptide over the crystal structure of MES bound in the active site of PP1. Catalytic resides are shown as sticks. MES is shown as green sticks, and the phosphorylated Ser6 residue of pRb-Tat peptide is shown as orange sticks. The pRb-Tat peptide is shown as orange ribbon. C. 1H4 inhibits kinetics of pRb-Tat peptide dephosphorylation by PP1α. Recombinant PP1α was assayed with pRb-Tat (WT or QACA mutant, 120 µM) in the absence or presence of 1H4 as indicated. The reactions were stopped at indicated time points and the phosphate release was quantified by malachite green assay. Initial velocity was calculated by linear regression in Prism. D. 1H4 inhibits kinetics of pRb-cdNIPP dephosphorylation by PP1α. Recombinant PP1α was assayed with pRb-cdNIPP1 in the absence or presence of 1H4 as indicated. Mutant pRb cdNIPP1 pA-RATA was used as negative control. The reactions were stopped at indicated time points and the phosphate release was quantified by malachite green assay. Initial velocity was calculated by linear regression in Prism. E and F. 1H4 competitively inhibits pRb-Tat peptide dephosphorylation by PP1α. Initial rates of pRb-Tat peptide dephosphorylation by PP1α were assayed at the indicated concentrations of the substrate in the absence or presence of 300 µM 1H4 or non-HIV-1 inhibitory 1G3. The amount of the released phosphate was quantified with malachite green. The V_MAX_ and Km were calculated by non-linear regression analysis in Prism with the assumption that 25% of the substrate contained the phosphate group. Transformation of the data to Lineweaver-Burk plot (panel E) showed competitive inhibition of pRb-Tat dephosphorylation.

We previously showed that HIV-1 Tat binds to the RVxF pocket of PP1 *in vitro* using competition assays [6]. Here, we analyzed the effect of 1H4 on the binding of the Tat RVxF sequence to PP1 using hybrid PP1 substrates containing a substrate phosphopeptide linked to RVxF-containing sequences derived from Tat or NIPP1. As the substrate phosphopeptide, we used a retinoblastoma protein-derived HIPR(pS)PYKFPSSPLR peptide (pRb) that is efficiently dephosphorylated by PP1 but not by the enzymatically-related PP2A [13]. The pRb peptide was linked to an extended RVxF-containing sequence derived from Tat (KKCCFHCQVCFITK) (pRb-Tat peptide) or the central domain of NIPP1 (KRKRKNSRVTFSED). To visualize the interaction between pRb-Tat and PP1 we built a computational model of the pRb-Tat peptide with PP1 complex (described in Materials and Methods). Based on this model, the pRb-Tat peptide is able to bind to the RVxF- accommodating groove of PP1 and, at the same time, to reach the active site of PP1 without any tension (Fig. 5A, green spheres; Fig. 5B, gray sticks). The pRb-Tat peptide was efficiently dephosphorylated by PP1 *in vitro* (Fig. 5C, V_0_ = 0.31 µM·min^-1^). Similar dephosphorylation kinetics were observed for the pRb-NIPP1 peptide (Fig. 5D, Vo = 0.56 µM⋅min^−1^), but not for the mutant pRb-NIPP1 pA-RATA peptide (HIPR(pS)PYKFPSSPLRAAAAASRATASED) which was a very poor PP1 substrate (Fig. 5D, Vo = 0.004 µM⋅min^−1^). Interestingly, the dephosphorylation of the pRb peptide (HIPR(pS)PYKFPSSPL) was significantly slower than pRb-Tat or pRb-NIPP1 peptides (Vo = 0.014 µM·min^−1^, data not shown). The increased dephosphorylation of pRb-Tat and pRb-NIPP1 peptides suggests that the extended RVxF motif might accelerate the dephosphorylation reaction likely due to the binding to PP1 during the process of substrate recognition. Dephosphorylation of the pRb-Tat QACA peptide (HIPR(pS)PYKFPSSPLR KKCCFHCQACAITK) having a mutation in the RVxF sequence was significantly reduced (Fig. 5C, V_0_ = 0.17 µM·min^−1^). Addition of 1H4 at 3-fold molar excess (480 µM) over pRb-Tat (160 µM) inhibited pRb-Tat dephosphorylation and reduced the rate of dephosphorylation (Fig. 5C, V_0_ = 0.19 µM·min^−1^) to the rate of pRb-Tat QACA dephosphorylation (Fig. 5C, V_0_ = 0.17 µM⋅min^−1^). The addition of 1H4 also reduced the rate of pRb-cdNIPP1 phosphorylation (Fig. 5D, V_0_ = 0.37 µM⋅min^−1^). These observations suggest that 1H4 is likely to interfere with the interaction of the RVxF motif with PP1. To further investigate the effect of 1H4 on the dephosphorylation of the pRb-Tat peptide, we analyzed the initial velocity versus pRb-Tat peptide substrate concentration plots. Addition of 1H4 inhibited pRb-Tat dephosphorylation by increasing Km but not Vmax (Fig. 5D). In contrast, a non-HIV-1 inhibitory 1G3 compound did not inhibit pRb-Tat dephosphorylation but instead induced dephosphorylation as evidenced by the decreased Km (Fig. 5D). Visualization on a Lineweaver-Burk plot showed a competitive nature of pRb-Tat inhibition by 1H4 (Fig. 5E), which is evidenced by a common intercept on the 1/Vo axis. This was expected because the dephosphorylation site and the RVxF-containing sequence were fused into one hybrid substrate and 1H4 interferes with the binding of this substrate to PP1. Taken together, these results demonstrate that 1H4 interferes with the binding of the RVxF motif to PP1.

### 1H4 does not Inhibit Enzymatic Activity of PP1 in vitro

**Figure 6:**
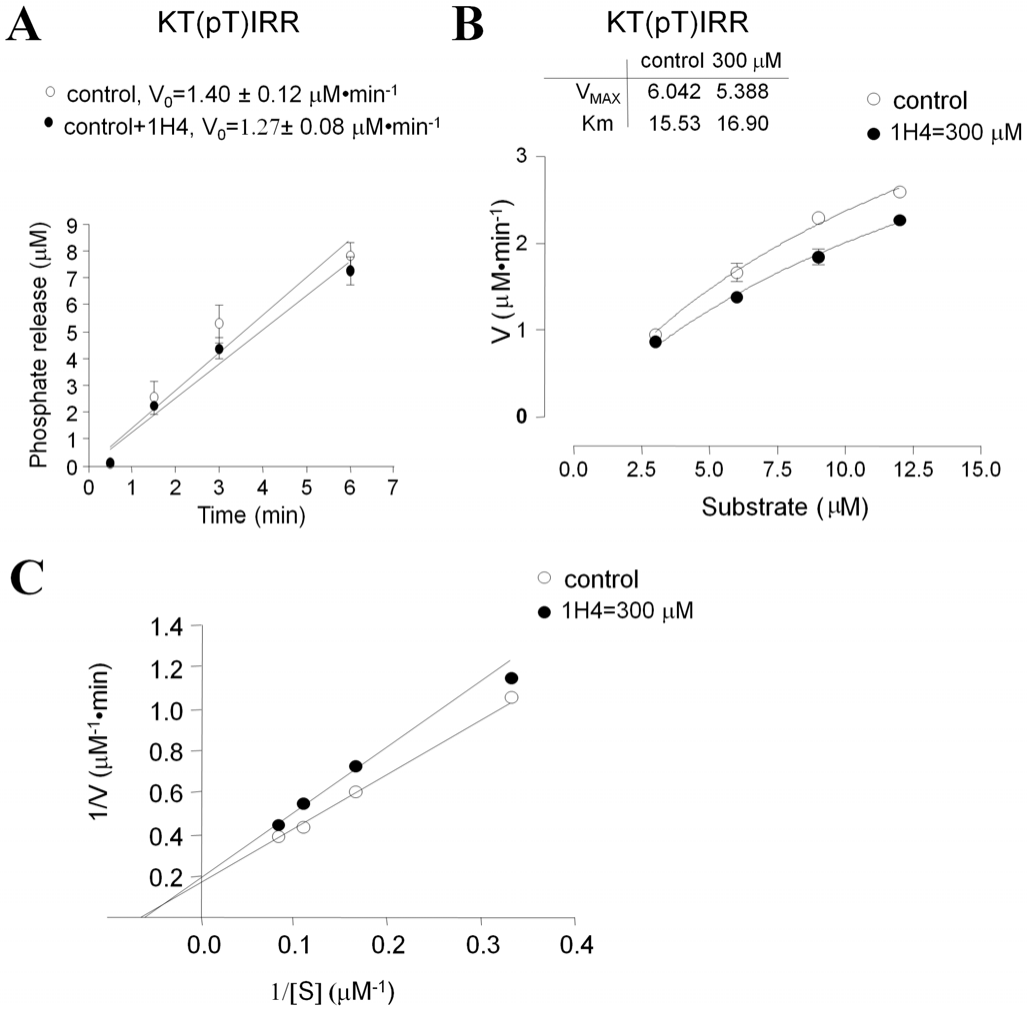
1H4 has no effect on PP1 enzymatic activity. A. 1H4 has no effect on the kinetics of KT(pT)IRR peptide dephosphorylation by PP1α. Recombinant PP1α (0.005 Units) was assayed with KT(pT)IRR peptide (3 µM) in the absence or presence of 1H4, and the reaction was stopped at indicated time points by the addition of malachite green solution. The amount of released phosphate was quantified by the absorbance and phosphate concentration was recalculated using standards. Initial velocity was calculated by linear regression in Prism. B and C. 1H4 has no effect on Km and V_MAX_ of KT(pT)IRR peptide dephosphorylation by PP1α. Initial rates of KT(pT)IRR peptide dephosphorylation by PP1α were assayed at the indicated concentrations of the substrate in the absence or presence of 300 µM 1H4. The amount of released phosphate was quantified with malachite green. The V_MAX_ and Km were calculated by non-linear regression analysis for Michaelis-Menten equation in Prism. The data were transformed to Lineweaver-Burk representation shown in panel C.

To determine whether 1H4 has an effect on the enzymatic activity of PP1, we used recombinant PP1α and a generic substrate, phosphorylated KT(pT)IRR peptide which is recognized equally well by PP1 and PP2A [13]. The KT(pT)IRR peptide (3 µM) was efficiently dephosphorylated by PP1α (Fig. 6A, V_0_ = 1.4 µM·min^−1^). Very little inhibition of PP1α activity was observed when 1H4 (300 µM) was added to the reaction (Fig. 6A, V_0_ = 1.3 µM·min^−1^). We further investigated the effect of 1H4 on PP1 enzymatic activity by analyzing the initial velocity versus KT(pT)IRR peptide substrate concentration plots in the absence and presence of 1H4 that were approximated by Michaelis-Menten equation (Fig. 6B) and also visualized in Lineweaver-Burk representation (Fig. 6C). The addition of 1H4 had minimal effect on Vmax and Km (Fig. 6B) further supporting the conclusion that 1H4 has no direct effect on PP1 enzymatic activity.

### 1H4 Prevents the Intracellular Interaction of Tat with PP1

**Figure 7:**
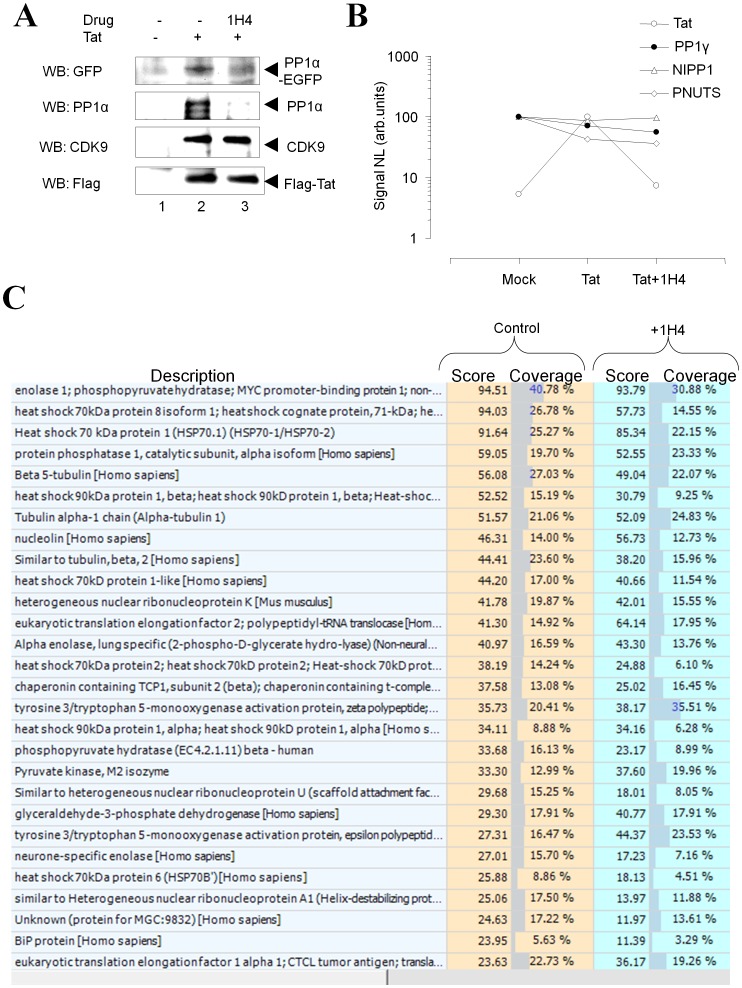
1H4 prevents the interaction of Tat with PP1 in cultured cells. A. Effect of 1H4 on PP1co-immunoprecipitation with Tat. 293T cells were transfected with Flag-tagged Tat and PP1α-EGFP. Flag-Tat was immunoprecipitated with anti-Flag antibodies from the cells extracts and probed with antibodies against EGFP to detect PP1and against Flag to detect Tat. Lane 1, untreated whole cell extract; lane 2, cells treated with 10 µM 1H4; lane 3, mock-transfected cells. B. 1H4 has no effect on PP1 association with NIPP1 and PNUTS. 293T cells were transfected with Flag-tagged Tat. PP1 was precipitated with microcystin agarose. The associated proteins were trypsinized and analyzed by nano-LC MS/MS. Liquid chromatography peak amplitudes for specific peptides derived from Tat (551.95 Da), PP1α (500.78 Da), NIPP1 (501.77 Da) and PNUTS (110.88 kDa) are shown, see details in text. The peptides were identified through MS/MS sequencing analysis by SEQUEST. C. Effect of 1H4 compound on a cell proteome. 293T cells were treated with 10 µM 1H4 for 18h or untreated and lysed as described in Materials and Methods. Lysates were trypsinized, fractionated by ion-exchange chromatography and then analyzed on by LC-MS-MS using C18 column. MS-MS data were analyzed by SEQUEST. The 28 major proteins having highest Score in SEQUEST are shown.

During HIV-1 infection, Tat facilitates PP1α translocation into the nucleus [6]. To analyze whether 1H4 disrupts the interaction of Tat with PPlα, we expressed PP1α-EGFP along with Flag-Tat, in the absence and presence of 1H4 (Fig. 7A). Flag-Tat co-precipitated with PP1α-EGFP (Fig. 7A, IP: α-Flag, lane 2) in accord with our previous report [6]. The addition of 10 µM 1H4 reduced the amount of PP1α-EGFP that co-precipitated with Tat (Fig. 7A, lane 3). Similarly, 1H4 reduced the association of Tat with endogenous PP1α as detected with PP1α-specific antibodies (Fig. 7A second panel, lanes 2 and 3). In contrast, 1H4 had no effect on the association of Tat with CDK9/cyclin T1 as shown by the equal presence of CDK9 (Fig. 7A, third panel lanes 2 and 3). Thus, 1H4 appears to interrupt the interaction between Tat and PP1α, without affecting the association of Tat with CDK9 thereby decreasing the amount of PP1 available to regulate HIV-1 transcription.

### 1H4 has no Effect on the Interaction of PP1 with NIPP1 and PNUTS

To analyze the specificity of the effect of 1H4, we analyzed the association of PP1 with the cellular regulatory subunits, NIPP1 and PNUTS, compared to the association with Tat. PP1 was precipitated on microcystin-sepharose from cell lysates and the precipitated proteins were trypsinized and analyzed by LC-MS/MS spectrometry. Figure 7B shows the relative amounts of Tat and PP1 subunits PNUTS and NIPP1 in different experiments. We used Normalization Level (NL) or the amplitude of MS peak signal as a value proportional to sample amount. First, the specific peptides were detected by SEQUEST. Then the exact mass and Retention Time of the peptides were used to filter the LC data. As previously shown, the LC-MS peak area could be used for sample amount quantification in a wide range of sample concentrations [17]. Our data were measured in the linear range of the NL signal. Figure 7B represents the amplitude of the LC peak for specific peptides of the following proteins: Tat (peptide RAPQDSQTHQASLSK, m/z  = 551.95 Da, z  = 3+), PNUTS (peptide GPQGPGGGGINVQEILTSIMGSPNSHPSEELLK, m/z  = 1100.88 Da, z  = 3+), NIPP1 (peptide VFLIDLNSTHGTFLGHIR, m/z  = 510.77 Da, z  = 4+) and PP1α (peptide LNLDSIIGR, m/z  = 500.78, z  = 2+). The amplitude of the signal was normalized for each peptide to its maximum on the samples set. PNUTS and NIPP1 were equally associated with PP1 in mock-transfected, Tat-transfected and Tat-transfected cells treated with 1H4 (Fig. 7B). In contrast, Tat association was reduced in the Tat-transfected cells that were treated with 1H4 (Fig. 7B). Therefore, 1H4 affected the interaction of PP1 with Tat without any effect on the interaction of PP1 with PNUTS or NIPP1.

### 1H4 has no Effect on the Expression of Cellular Proteins

To determine if 1H4 has a negative effect on protein expression profiles, we analyzed the global cellular proteome by mass spectrometry. Protein expression was analyzed in 293T cells untreated or treated with 1H4 as described in Materials and Methods. We detected expression of 1722 proteins in the untreated sample and 1739 proteins in the 1H4-treated sample (not shown). Analysis of the 28 proteins having the highest Scores in SEQUEST search showed very close score (credibility of search result) and coverage (part of the database protein sequence found experimentally) (Fig. 7C) indicating that 1H4 treatment did not significantly change the cellular proteome.

### 1H4 Prevents PP1 Translocation to the Nucleus

**Figure 8:**
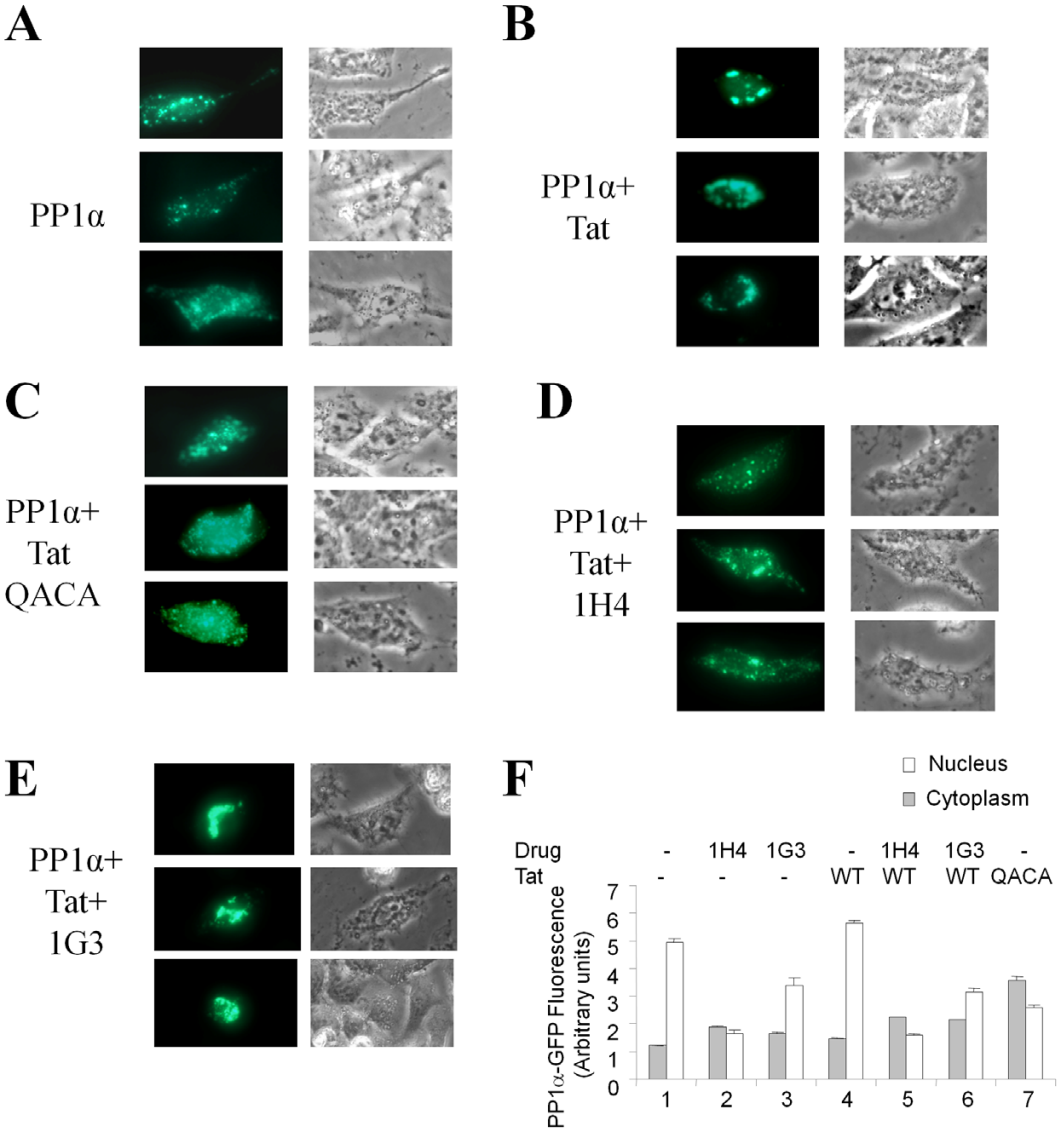
1H4 compound disrupts the Tat-mediated translocation of PP1 into the nucleus. HeLa cells were transfected with PP1α-EGFP (PP1α) (A), PP1α-EGFP and WT Flag-Tat (B, D and E) or PP1α-EGFP and Flag-Tat ^35^QACA^38^ mutant (C) and treated with 10 µM 1H4 (D) or control 1G3 compound (E) for 18 hours. The cells were photographed on Olympus IX51 using a blue filter for EGFP fluorescence or phase contrast with 600X magnification. F, 293T cells were transfected with PP1α-EGFP or PP1α-EGFP and WT Tat or Tat QACA mutant expression vectors. At 24 hrs posttransfection cells were lysed in low salt buffer and cytoplasmic extract was separated from the nuclear material by centrifugation. Fluorescence was measured in the nuclear and cytoplasmic fractions using Perkin-Elmer Luminoscan.

In live cells, PP1α is dynamically distributed between the cytoplasm and the nucleus, and its shuttling into the nucleus is thought to be regulated by its interaction with sds22 and inhibitor-3 regulatory subunits [18]. We previously showed that HIV-1 Tat facilitated nuclear localization of PP1α via an effect that requires the intact QVCF sequence of Tat [6]. We analyzed the effect of 1H4 on nuclear localization of PP1α in HeLa cells that were transfected with Flag-Tat and PP1α-EGFP expression vectors and treated with 1H4 or a control compound for 18hrs. In untreated cells, PP1α was localized in the perinuclear area and cytoplasm (Fig. 8A). Co-expression of Tat increased nuclear and perinuclear localization of PP1α (Fig. 8B). Co-expression of mutant Tat ^35^QACA^38^ in contrast led to a more homogeneous distribution of PP1α (Fig. 8C). Treatment with 1H4 diminished nuclear localization of PP1α in the presence of Tat (Fig. 8D). In contrast, treatment with the inactive compound 1G3 led to a more pronounced nuclear PP1α localization in the presence of Tat (Fig. 8F). To achieve quantifiable results, we measured fluorescence of PP1α-EGFP in nuclear and cytoplasmic fractions of 293T cells that were transfected with PP1α-EGFP or PP1α-EGFP and Tat expression vectors and treated with 1H4. The cytoplasmic and nuclear fractions were separated as described in Materials and Methods. Analysis of EGFP fluorescence showed a significant decrease of nuclear PP1α-EGFP in the 1H4-treated cells compared to the untreated controls or the cells treated with 1G3 compound (Fig. 8F). Unexpectedly, this was observed both in the absence and the presence of Tat (Fig. 8F). Moreover, Tat has only moderate (10–20% in several independent experiments) effect on PP1 distribution to the nucleus. But strikingly, Tat QACA mutant expression resulted in increased accumulation of PP1 in the cytoplasm (Fig. 8F, lane 7).

Taken together, our experiments showed that a small molecular mimetic of the RVxF motif efficiently inhibited HIV-1 transcription apparently by disrupting the interaction of Tat with PP1 and affecting the cellular distribution of PP1.

## Discussion

Our study identified a novel hit compound, 1H4 that inhibited Tat-induced transcription and HIV-1 replication at low micromolar concentrations. The minimal acridine core (9-amino-1,2,3,4-tetrahydroacridine) was not active as an HIV-1 transcription inhibitor (data not shown), suggesting that substitutions at positions 4 and 9 that are present in 1H4 compound (Fig. 4B) are important for the antiviral activity. According to the *in silico* binding model, the substitution at position 4 binds the Gln262 side chain of PP1 providing additional van der Waals interactions (Fig. 2, panel 1). The substitution at position 9 of the acridine core points along the narrow hydrophobic channel of PP1 toward Cys293 and Tyr255 (Fig. 2, panel 1). Flexible substitutions at position 9 are likely to be preferred. The extended 9-carboxoacteamide moiety of 1H4 provides sufficient flexibility to fit the groove’s shape and bridge guanidine of Arg261 and carbonyl Cys291 with a hydrogen bond network (Fig. 4A). Further modifications and analysis of the activity of the compounds with extended flexible chains at the 9^th^ position will likely generate additional active inhibitory compounds. On the other hand, branched substitutions at the 9^th^ position would likely abort formation of the hydrogen bond network and render compounds inactive.

Previously reported inhibitors of HIV-1 transcription targeted TAR RNA, Tat and host cell factors involved in HIV-1 transcription (see for details [19]). The fluoroquinoline derivative, K-37, interacted with TAR RNA *in vitro* and inhibited Tat-induced transcription as well transcription induced by other artificial RNA- dependent transcriptional activators [20]. A structural analog of K-37, the 6-aminoquinolone derivative, WM5, efficiently bound TAR RNA *in vitro* with nanomolar affinity and inhibited HIV-1 replication in acutely infected and chronically infected cells [21], [22]. Compound 3, which was developed from the WM5 lead compound, inhibited HIV-1 replication in acutely and chronically infected T cells and macrophages and also inhibited Tat-induced transcription although at 5-10 fold higher concentrations [23]. Whether compound 3 targets TAR RNA remains to be seen. Other HIV-1 inhibitory compounds that targeted TAR RNA included 2'-O-methyl (OMe) oligonucleotide mixmers or oligonucleotides containing tricyclo-DNAs [24], amino disaccharides with an alpha-(1→4) linkage that inhibited binding of Tat to TAR RNA at subnanomolar concentrations [25], substituted purines containing a side chain with a terminal amino or guanidyl group [26] and isoquinoline derivatives bearing guanidinium group or amino group-terminated side [27].

In addition to targeting TAR RNA and Tat, host cell factors involved in the regulation of HIV-1 transcription were also considered as potential targets. Tat recruits the human transcriptional coactivator PCAF (p300/CREB binding protein-associated factor) that binds to Tat acetylated at lysine 50 and facilitates transcription of the integrated HIV-1 provirus (reviewed in [28]). The PCAF-targeted N1-aryl-propane-1,3-diamine based lead “compound 2″ inhibited the binding of GST-PCAF BRD to Tat acetylated on lysine 50 [29] and also inhibited HIV-1 transcription [30]. A lead N-aminoimidazole derivative NR-818 inhibited HIV-1 transcription by prolonging the binding of NF-κB to its consensus sequence and also increased the acetylation of histones H3 and H4 within the nucleosome nuc-1 at the transcription initiation site [31]. In the present study, we targeted PP1 that we previously showed to be critical to Tat-dependent HIV-1 transcription [5], [6]. In cultured cells, HIV-1 transcription is inhibited by the expression of NIPP1, a nuclear regulatory subunit of PP1 [5], and by okadaic acid which inhibits PP1 activity [32]. HIV-1 transcription is also decreased by the expression of a catalytically inactive mutant of PP1 (PP1γD64N) [6]. The current study extends our previous findings and demonstrates that the inhibition of HIV-1 can be achieved through the targeting of the interaction of PP1 and Tat with small molecules. While our immunofluorescence results are consistent with the previous finding that HIV-1 Tat facilitates translocation of PP1 to the nucleus, direct fluorescence measurement showed that 1H4 has an effect on PP1 distribution in the absence of Tat and that Tat has only moderate effect on PP1 localization. There was however a striking difference in the effects of WT Tat and Tat QACA mutant that showed increased PP1α in the cytoplasm of HeLa cells (Fig. 8C) or 293T cells measured by direct fluorescence (Fig. 8F) suggesting that Tat’s QVCF sequence interacts with PP1 in cultured cells. The Tat-independent effect of 1H4 on PP1 can explain inhibition of basal HIV-1 transcription (Fig. S2B) that could be due to the disruption of the interaction of PP1 with a shuttling regulatory subunit such as sds22 and inhibitor-3 that form a sandwich with inactive PP1 to shuttle it into nucleus [18]. We are currently in the process of further detailed analysis of the effect of PP1-targeted small molecule compounds on PP1 shuttling in the absence and presence of Tat.

There are several possibilities for how PP1 affects HIV-1 replication. First, a possible biological target of PP1 could be CDK9 [13], [32]. CDK9/cyclin T1 associates with 7SK RNA [33], [34] and a hexamethylene bisacetamide (HEXIM1) protein [35], [36] and this interaction inhibits the activity of CDK9 [33], [34], [35], [36] Recently, PP1α and protein phosphatase 2B (PP2B) were shown to disrupt the interaction between CDK9/cyclin T1 and 7SK RNA/HEXIM1, thereby inducing the activity of CDK9/cyclin T1 [12] Currently, the regulatory subunit that targets PP1 to CDK9 remains unknown, although in HIV-1 infection, the viral Tat protein apparently serves this role. In this regard, based on the finding that enhanced PP1 binding by the Tat Q35R mutant disrupts the interaction of Tat with CDK9/cyclin T1 [6], it is likely that Tat interacts first with PP1 and then CDK9/cyclin T1, possibly releasing PP1 to dephosphorylate CDK9 leading to its dissociation from 7SK RNA.

The transcription factor Sp1 is required by HIV-1 for a basal transcription [37] and can target cyclin T1 to the LTR in the absence of Tat or TAR RNA [38]. An increase in Sp1 phosphorylation induced by Tat and DNA-PK enhances HIV-1 transcription [39]. A possible second biological target for PP1-dephosphorylation could be Sp1, which is found associated with PP1 at cellular promoters [40].

Alternatively, RNA polymerase II could be a third PP1 substrate. We have previously shown that the C-terminal domain of RNA polymerase II is indeed dephosphorylated by PP1 [4]. Our recent study showed that NIPP1 can serve as an RNAPII targeting subunit of PP1 [41] and thus altered RNAPII dephosphorylation could also be considered as a potential inhibitory mechanism. Interestingly, stable or transient expression of cdNIPP1 does not affect the viability of cells even though the association of CDK9 with 7SK RNA is increased [5]. The expression of cdNIPP1 disrupts the interaction of PP1 with Tat and inhibits HIV-1 [5]. Recently, a combined approach of in silico screening and a multistep biochemical validation procedure identified many novel PP1 interactors that contained the novel PP1 binding motifs, "SILK" and "MyPhoNE" [8]. Thus, small molecule compounds that disrupt the interaction of PP1 with these novel PP1-binding motifs and also with the RVxF motif, might deregulate subsets of cellular PP1 holoenzymes and selectively target viral or cellular pathways.

Further investigation is required to elucidate fully the physiologically relevant PP1-cell factor interaction(s) for HIV-1 replication. Nevertheless, to the extent that such an interaction is critical to the propagation of HIV-1 in human cells, our proof-of-concept finding of a non-cytotoxic PP1-inhibitor is an important advance that potentially broaches a new class of anti-HIV drugs. 1H4 is the first example of a small molecule inhibitor of PP1 that affects HIV-1 transcription.

Our findings open PP1 as a new avenue for the design of novel antiretroviral therapeutics. A similar approach was recently proposed for the design of modulators of PP1 that target various regulatory binding sites including the RVxF-binding site, which may complement existing drugs that target protein kinases [42].

## Materials and Methods

### Materials

Human endothelial kidney 293T (293T) and CEM cells were purchased from ATCC (Manassas, VA). CEM-HIV-1 (LTR) GFP cells (courtesy of Dr. Jacques Corbeil) were obtained from the NIH AIDS Research and Reference Reagent Program. All cells were cultured at 37°C and 5% CO_2_ **.** CEM-GFP cells were cultured in RPMI Medium 1640 containing 10% fetal bovine serum (FBS), with 1% antibiotic solution (penicillin and streptomycin) and 500 µg/ml G418 (Invitrogen). 293T and HeLa cells were cultured in Dulbecco's Modified Eagles Medium (DMEM) containing 10% fetal bovine serum (FBS) (Gibco-BRL) and 1% glutamine (Invitrogen).

The HIV-1 reporter contained HIV-1 LTR (−138 to +82) followed by a nuclear localization signal (NLS) and the Lac Z reporter gene (courtesy of Dr. Michael Emerman, Fred Hutchinson Cancer Institute, Seattle, WA) and the pNL4-3.Luc.R^-^E^-^ (courtesy of Dr. Nathaniel Landau) were obtained from the NIH AIDS Research and Reference Reagent Program. PP1α-EGFP and Flag-Tat expression vectors were previously described [3]. Antibodies for Flag epitope and α-tubulin were purchased from Sigma (Atlanta, GA). Protein G agarose was purchased from Upstate (Lake Placid, NY). Antibodies against PP1α were purchased from EMD Chemicals (Gibbstown, NJ).

### Virtual Screening Procedure

Enamine (Kiev, Ukraine) stock collection refers to off-the-shelf collection of screening compounds. Currently, Enamine's screening collection is divided into three (Historical, Screening and Advanced collections (http://www.enamine.net/index.php?option=com_content&task=view&id=22) but by the time of the study was initiated, it was one generic compound collection that was used as a source for the virtual screening. Instant JChem was used for structure database management, search and data mining (ChemAxon, Budapest, Hungary; http://www.chemaxon.com). Schrodinger’s (New York, NY; www.schrodinger.com) modules LigPrep and Qikprop were used to generate 3D conformations of chemical structures and to predict ADMET properties, respectively. Docking experiments were done with QXP package [43] and Glide of Schrodinger. Chimera [44] and lzm from QXP were used for the visual analysis. Docking studies were performed on Linux dual core AMD workstations. Compound structures were converted to 3D stereo-conformers generated and geometrically optimized with Ligprep module of Schrodinger. If a molecule contained more than two stereo-centers it was excluded from the study. Before the docking stage the compound database (∼600K at that moment) was pre-processed to exclude structures with reactive groups. Then Lipinski’s Rule of Five was employed to produce drug-like stock (∼300,000) with the only exception of MW ranging from 280 to 550. The shift toward heavier molecules was made to increase the potential number of molecules with sufficient contact surface. Coordinates of the receptor molecule, PP1, were processed with pdb2mm module of the QXP package. Binding site was defined as PP1 residues within the sphere of 1.2 nm with center as CG atom of Leu243. Sdock routine of QXP docking engine was employed at 100 steps per ligand and 5 conformations per complex were saved.

### Processing of Docking Results

Docking results were processed sequentially in two steps, a first step rough filtering was followed by the second step fine geometric filtering. For the rough filtering we used scoring values generated by QXP whereas for the fine geometric filtering we employed geometry based filters as described below. A program for geometric filtering, Multifilter, was developed in-house.

#### Step one, rough filtering

Scoring values were calculated by qxp+ and included pI, represents binding energy (pI = -log10Ki); Cntc, distance dependent ligand-protein contacts (Kj/Mol); Intl, estimation of ligand's strain, entropy, internal contacts (Kj/Mol); and Hbnd, distance dependent hydrogen bond function (Kj/Mol). Compounds with pI >4, Cntc < −55, Intl <8 Hbnd < -3 and Intl <7 have passed first filtering step. Next, we selected compounds that had close contacts with the methyl group of Leu243 and the phenyl ring of Phe257 of PP1. A distance filter of 4.3 Å from any ligand's carbon atom to CD1 of Leu243 and CZ of Phe257 (PDB nomenclature) were applied. The distance of 4.3 Å was selected to account for PDB resolution (3 Å) as well as possible calculation inaccuracies.

#### Step two, geometric filtering

During the step one rough filtering we identified 4 distinct binding modes. In the first mode, compounds that filled the region near Tyr255 were selected by applying a distance cut-off of 4 Å from the tyrosine’s phenolic oxygen. In the mode two, compounds that bound in a similar fashion to the RRVSFA peptide and that were within 6.5 Å from Cβ of Asp166 were selected. The third mode included compounds that were within 4 Å of the amide oxygen of Gln262, and the fourth mode extracted compounds that were confined to the Val66’ and Phe68’ hydrophobic sub-sites and formed extensive hydrogen bonds with at least two of the following residues: Lys260, Arg261, Asp242, Val 289, M290 and Cys291. Filtering according to the first mode yielded 117 compounds; the second mode –51 compounds; the third mode –80 compounds; and the fourth mode –43 compounds. After removing the duplicates (i.e. compounds that fit into more than one model), we obtained 262 unique compounds that were further evaluated biologically for inhibition of HIV-1.

### Modeling of pRb-Tat PP1 Complex

Coordinates from a crystal structure of PP1 bound to Spinophilin (PDB ID: 3EGG) [45] were used for the reference in the RVxF binding site (hydrophobic groove). To model the complex with pRb-Tat, the conformation of RVxF motif of pRb-Tat peptide (residues Val25-Phe27) was manually adjusted to mimic that of Spinophilin (residues Ile449-Phe451). Then conformation of pRb-Tat peptide was energetically minimized with a distance restrain (4 Å) between phosphorus atom of phosphorylated Ser6 of the pRb-Tat peptide and manganese atom in the active site. Coordinates of all PP1 atoms and Val25-Phe27 of pRb-Tat were kept frozen to prevent any disturbance during energy minimization. Minimization procedure was performed with MacroModel module of Schrodinger. OPLS2005 force filed was used to conduct 2000 steps of PRCG protocol in implicit solvent environment. During the minimization the pRb-Tat peptide made a turn and followed the acidic groove of PP1 toward the active site. In the resulted complex the sulfoxyl group of the phosphorylated Ser6 from pRb-Tat reached a position close to that of 2-(N-morpholino)-ethanesulfonic acid (MES) in the reference 3EGG complex. The final complex was subjected another 500 steps of energy minimization with all atoms allowed to move.

### Tat-induced HIV-1 Transcription in CEM-GFP Cells

The E1-deleted recombinant Adenovirus carrying Tat was generated as previously described [3], [46]. CEM-GFP cells were infected with Ad-Tat in 96-well plates containing 400,000 cells/well. After 24 h of incubation at 37°C, 10 µL aliquots were removed, supplemented with trypan blue and counted to determine cellular viability. The remaining cells were transferred to a white plate (Perkin-Elmer) and fluorescence was measured with 480 nm excitation and 510 nm emission on Luminescence Spectrometer LS50B (Perkin-Elmer). To measure toxicity, propidium iodide (Sigma) was added at 250 µg/ml to the cells for 30 min at 37^o^C. The propidium iodide fluorescence was measured at 488 nm excitation and 617 nm emission on the Luminescence Spectrometer as described above.

### Transient Transfections

293T cells were co-transfected with Tat-expressing vector and HIV-1 LTR-L*acZ* and CMV-EGFP at 30% confluence using lipofectamine and Plus reagents (Invitrogen). After transfection, the cells were cultured for additional 48 h, and β-galactosidase activity was analyzed as previously described [16]. Transfections were normalized using the EGFP fluorescence. HeLa cells were transiently co-transfected with plasmids expressing PP1α-EGFP, Flag-tagged WT Tat or Tat V36A/F38A (Tat QACA) mutant using lipofectamine and Plus reagents. After transfection, the cells were treated with the indicated PP1-targeted compounds overnight. Expression of PP1α-EGFP was detected on fluorescent microscope Olympus IX51 using a blue filter for EGFP fluorescence or phase contrast and photographed at 600x magnification.

### Inhibition of HIV-1 Replication using PP1-targeted Small Molecule Compounds

To prepare pNL4-3 virus, HeLa cells were transfected with pNL4-3 genomic clone using the lipofectamine method. After 72 h post-transfection, media was collected, and supernatant virus was quantified by reverse transcriptase assay. MT4 cells were grown to 70% confluence and inoculated with pNL4-3 virus. Subsequently, the infected cells were treated with 1H4 or 1G3 compounds and media samples were collected at the indicated time points. The activity of reverse transcriptase (RT) was determined in the supernatants (10 µl) incubated in a 96-well plate with RT reaction mixture containing 1x RT buffer (50 mM Tris-HCl, 1 mM DTT, 5 mM MgCl_2_, 20 mM KCl), 0.1% Triton, poly(A) (10^–2^ U), poly(dT) (10^–2^ U), and (^3^H)TTP. The mixture was incubated overnight at 37°C, and 5 µl of the reaction mix was spotted on a DEAE Filtermat paper, washed four times with 5% Na_2_HPO_4_ and three times with water, and then dried completely. RT activity was measured in a Betaplate counter (Perkin Elmer).

### Dephosphorylation Assays

Malachite green dephosphorylation assays were carried out with the Ser/Thr phosphatase assay kit (Upstate, Lake Placid, NY) using recombinant PP1α (New England Biolabs, Ipswich, MA). In competition assays, about 0.005U of PP1α was incubated with KT(pT)IRR peptide (Upstate, Lake Placid, NY) or pRb (HIPR(pS)PYKFPSSPL)-linked peptides (New England Peptide, Gardner, MA). The following pRb peptides were used in the study: pRb-Tat, HIPR(pS)PYKFPSSPLRKKCCFHCQVCFITK; pRb-Tat QACA, HIPR(pS)PYKFPSSPLRKKCCFHCQACAITK; pRb-NIPP1, HIPR(pS)PYKFPSSPLRKRKRKNSRVTFSED; and pRb-NIPP pA-RATA, HIPR(pS)PYKFPSSPLRAAAAASRATASED. The reactions were carried out in PP1 reaction buffer (50 mM Tris-HCl pH 7.5, 100 mM NaCl, 2 mM dithiothreitol, 0.1 mM EGTA, 0.025% Tween-20) supplemented with 1 mM MnCl_2_ (New England Biolabs) in 25 µl reaction volume with the indicated concentrations of 1H4 or 1G3. In competition assay, 300 µM 1H4 was used. At indicated time points, 25 µl aliquots were removed and mixed with 100 µl of Malachite Green solution (Upstate). Absorbance of malachite green was determined at 620 nm and the phosphate concentration was recalculated using a calibration curve of phosphate standards prepared using 1 mM KH_2_PO_4_ solution.

### Co-immunoprecipitation and Western Blot

293T cells were transfected with PP1α-EGFP and Flag-Tat expression vectors, and further cultured for 48 h. The whole cell extracts were prepared as described previously [6]. About 1 mg of whole cell extract was supplemented with 3 µg of anti-Flag antibodies. Protein G-agarose beads were preblocked with 5% BSA and suspended in TNN buffer (50 mM Tris-HCl (pH 7.5), 0.5% NP-40, 150 mM NaCl) and the reaction was incubated in TNN buffer at 4°C for 2 h with rocking. The beads were precipitated and washed once with TNN buffer and once with the SDS-PAGE stacking buffer (25 mM Tris-HCl pH 6.8). The pellet was resuspended in 1X SDS loading buffer (25 mM Tris-HCl (pH 6.8) 4% SDS, 10% glycerol, 5% 2-mercaptoethanol, 0.002% bromophenol blue) and heated at 90°C for 3 minutes. The proteins were resolved on 12% SDS Tris-Tricine PAGE, to detect PP1α and Tat, and immunoblotted with indicated antibodies. About 10 µg of total protein was used for the input.

### Affinity Precipitation of PP1 using Microcystin-sepharose

293T cells were transfected with Flag-Tat expressing plasmid or mock-transfected with lipofectamine-Plus reagents followed by the treatment with two concentrations of 1H4 compound. At 18 hrs post transfection the cells were harvested, washed, lysed in a whole cell lysis buffer (50 mM Tris-HCl, pH 7.5, 500 mM NaCl, 1% NP-40, protease inhibitors cocktail) and used to precipitate endogenous PP1 on microcystin-sepharose (Millipore, Billerica, MA) as an affinity sorbent. The reaction was carried on in 50 mM Tris-HCl buffer, pH 7.5, 100 mM NaCl, 1% NP-40 with protease inhibitors cocktail for 3 hours at 4^o^C rotating. The beads were then washed with the precipitation buffer three times and once with AmBic buffer (50 mM ammonium bicarbonate, pH 8.0). Then 1 µg of Gold Trypsin (Promega) in 100 µl of AmBic was added to the beads and the reaction was incubated on a shaker overnight at 37^o^C. The supernatant was collected, dried on a SpeedVac and peptides were reconstituted in MS-LC grade water with 0.1% TFA and purified using Zip tips (Millipore) and manufacturer recommendations. The peptides were eluted from Zip tips in 30 µl of 80% acetonitrile with 0.1% TFA and dried on SpeedVac. The samples were reconstituted in MS-LC grade water containing 0.1% formic acid and loaded onto nano-LC for a mass spectrometry analysis.

### Mass Spectrometry Analysis

Samples were separated by reversed-phase liquid chromatography (HPLC), using micro-capillary column C18, coupled in line with nanospray and tandem mass spectrometer Thermo LTQ Orbitrap XL. The LC gradient was run for 60 min from 2% to 30% of acetonitrile containing 0.1% formic acid at flow rate 400 nL/min. In single measurement block we performed 1 FT MS scan and 3 data dependent FT MS/MS scans on major multi-charged MS peaks with resolution 30000. The normalized collision-induced dissociation (CID) energy was 35%. The MS/MS spectra were compared against those in the NCBI human protein database. Proteins that were not present in the database, i.e. recombinant Flag-Tat, were manually added to the database. Only peptides having X-correlation (*X*_corr_) cutoffs of 1.9 for [M +2H]^2+^, 2.3 for [M +3H]^3+^ and higher charge state were considered. These SEQUEST criteria thresholds resulted in less than 1% of False Discovery Rate. The proteome analysis of the spectra was made by Proteome Discoverer 1.2 software (Thermo Fisher Scientific).

### Cellular Proteome Analysis

293T cell at 50% of confluence were treated with 10 µM 1H4 or 1G3 compounds diluted in DMEM complete media for 18 hours. As a negative control untreated cells were used. Cells were lyzed in whole cell lysis buffer (50 mM Tris-HCl, pH 7.5, containing 0.5 M NaCl, 1% Nonidet P-40, 0.1% SDS and protease inhibitors cocktail (Sigma). The lysates were centrifuged at 20,000x g for 30 min at 4^o^C. The proteins from the supernatant were precipitated with cold acetone, centrifuged, dried and resuspended in 100 mM ammonium bicarbonate buffer, pH 8.0. Then the proteins were reduced and alkylated using DTT and iodoacetamide respectively. Reduced and alkylated proteins were trypsinized overnight at 37^o^C with 1 µg of trypsin used per 200 µg of protein used for reaction. The trypsinized samples were purified using C18 Zip tips. The samples were separated on SCX column using the NaCl gradient from 0 to 500 mM. Each collected fraction was subjected to LC-MS-MS procedure, described above. The MS-MS data were analyzed by SEQUEST.

### PP1α-EGFP Fluorescent Photographs

HeLa cells at 50% of confluence were transfected with PP1α-EGFP expressing plasmids using Lipofectamine LTX. The cells also were co-transfected with Flag-Tat and Flag-Tat V36A/F38A mutant expressing plasmids. As a negative control, we used HeLa cells treated with Plus/Lipofectamine LTX mix without adding a plasmid. After transfection the cells were treated with the compounds 1H4 and 1G3 at 10 µM concentration. After 18 hours of incubation cells were washed with PBS, observed and taken pictures on a phase contrast/fluorescent microscope Olympus IX51. Expression of PP1α-EGFP was detected on the microscope using a blue filter for EGFP fluorescence or phase contrast and photographed at 600x magnification.

### Nuclear and Cytoplasmic Distribution of PP1

Nuclear and cytoplasmic extracts were prepared using a modification of previously described method [47]. 293T cells were washed with PBS, scrapped into ice-cold PBS and precipitated by centrifugation. The cells were resuspended in ice-cold homogenization buffer (10mM Tris-HCl, pH 7.8, 6 mM MgCl_2_, 80 mM, KCl, 2 mM DTT, 250 mM sucrose, 0.1 mM EDTA) containing protease inhibitors (Sigma, St. Louis, MO) and then lysed by the addition of Triton X-100 to 0.1% (vol/vol) for 10 min on ice. The lysates were centrifuged at 10,000xg for 10 min at 4^o^C, and supernatants containing the cytosolic fraction were removed. The pellets containing nuclear proteins were resuspended in a whole cell lysis buffer (50 mM Tris-HCl, pH 7.5, 500 mM NaCl, 1% NP-40, protease inhibitors cocktail). Fluorescence was measured in cytoplasmic and nuclear lysates at 480 nm excitation and 510 nm emission on Luminescence Spectrometer LS50B (Perkin-Elmer).

### Inhibition and Enzymatic Data Analysis

Inhibition data were analyzed using GraphPad Prism 4 software (GraphPad Software, La Jolla, CA). The IC_50_ values were determined from a sigmoidal dose-response (variable slope) curve using four parameter logistic equation Y = Bottom+(Top-Bottom)/(1+1(logEC50-X)*Hill Slope). Dephosphorylation of chimeric substrates by PP1 was analyzed using GraphPad Prism 4 software. Enzymatic velocity vs. substrate concentration plots were fit using built-in enzyme kinetics for non-linear regression analysis. For the analysis of pRb-Tat dephosphorylation, we assumed that only 25% of the substrate contained phosphatase group and was active in the dephosphorylation. After determination of V_MAX_ and Km, data were transformed to create Lineweaver-Burk plots and lines were added using V_MAX_ and Km determined by non-linear regression analysis as described in a Prism instruction manual.

## Supporting Information

Figure S1 **Flowchart of** ***in silico*** **screening of PP1 inhibitors. Stage one,** analysis, pharmacophore model development and high throughput docking. Stage two (light gray), rough filtering of resulted complexes. Stage three (dark gray), fine filtering in parallel with four binding mode hypothesis.
(PDF)

Figure S2 **Effect of 1H4 on HIV-1 transcription, toxicity and CMV transcription.** A. Inhibition of HIV-1 transcription and toxicity of PP1 inhibitors in CEM-GFP cells. CEM-GFP cells were infected with Adeno-Tat and then treated with the indicated concentrations of the PP1 inhibitors for 24 h. GFP fluorescence was measured in live cells. The cells were supplemented with propidium iodide (PI), and its fluorescence was measured. B. Effect of 1H4 on HIV-1 and CMV transcription. HEK293T cells were transfected with HIV-1 LTR-LacZ in the absence or presence of Tat expression vector (Tat). The cells were also co-transfected with CMV- EGFP-expression vector and treated with 10 µM 1H4 (lane 2) or 10 µM 1G3 (lane 3). Twenty-four hours after transfection, the cells were lysed and first analyzed on a luminescence spectrometer (LS50B, Perkin-Elmer) with an attached 96-well plate scanner at 480 nm excitation and at 510 nm emission for EGFP, and then analyzed for β-galactosidase activity using ONPG as a substrate.
(PDF)

Table S1 **Analysis of 262 small molecules for the inhibition of HIV-1 transcription in CEM GFP cells infected with Ad-Tat.** Percent of inhibition is shown. Compounds chosen for further analysis are shown in gray.
(PDF)
