# Supplementary figures and images for: Small Molecules Targeted to a Non-Catalytic “RVxF” Binding Site of Protein Phosphatase-1 Inhibit HIV-1
Source: PLoS One. 2012 Jun 29;7(6):e39481. doi: 10.1371/journal.pone.0039481 (PMC3387161; doi:10.1371/journal.pone.0039481)

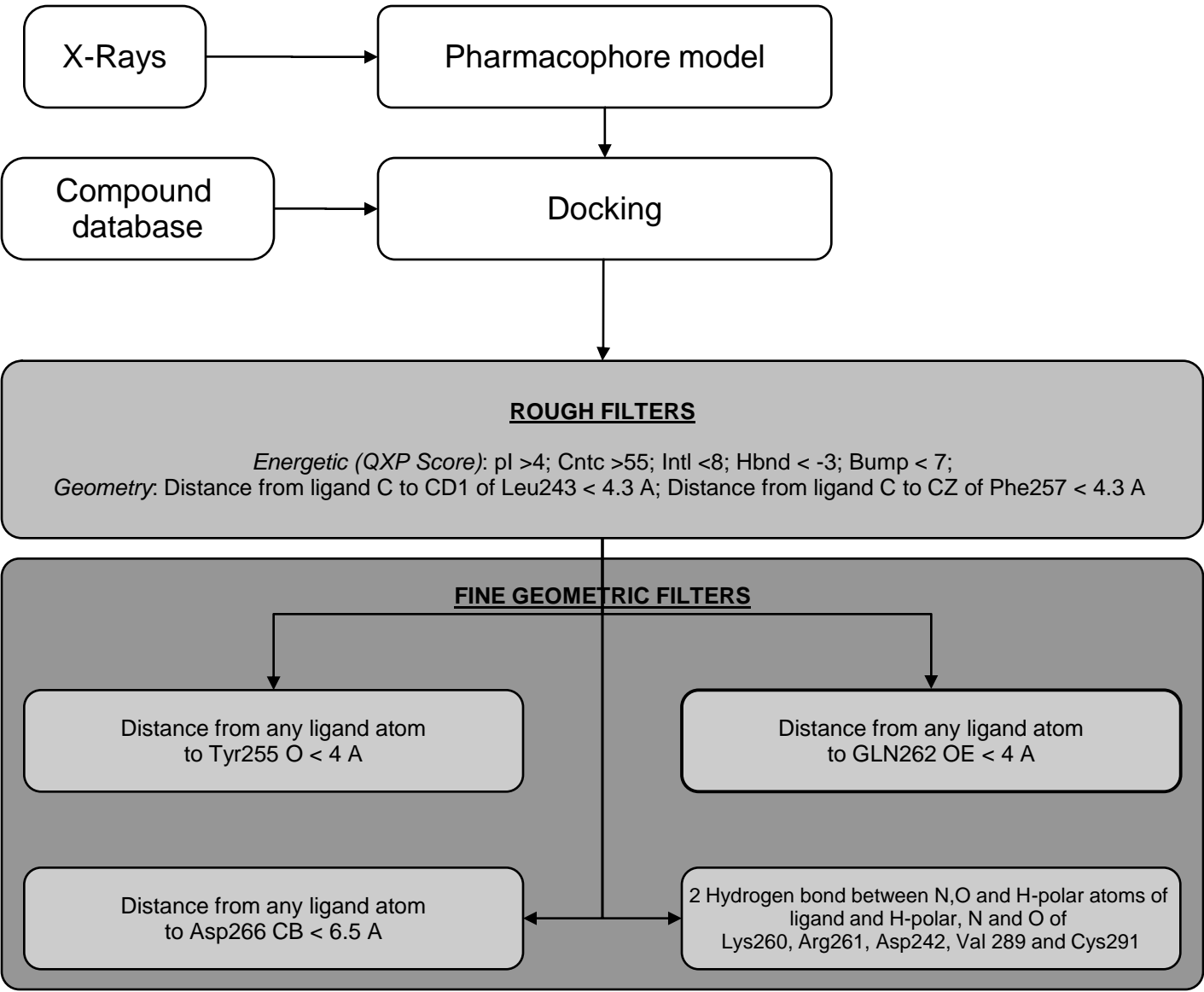

Supplement: Figure S1 — Flowchart of in silico screening of PP1 inhibitors. Stage one, analysis, pharmacophore model development and high throughput docking. Stage two (light gray), rough filtering of resulted complexes. Stage three (dark gray), fine filtering in parallel with four binding mode hypothesis. (PDF) [file pone.0039481.s001.pdf]

**A**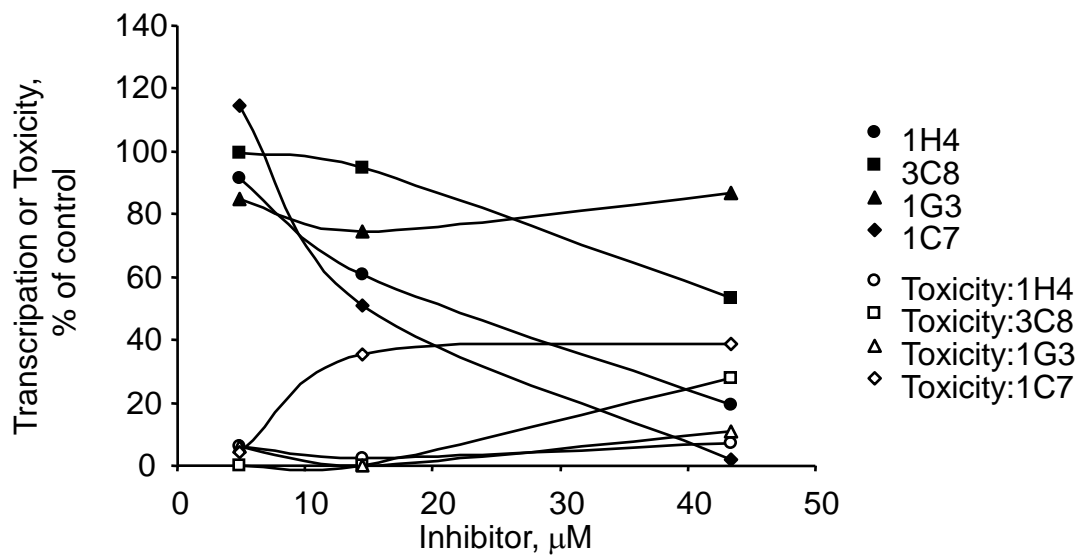**B**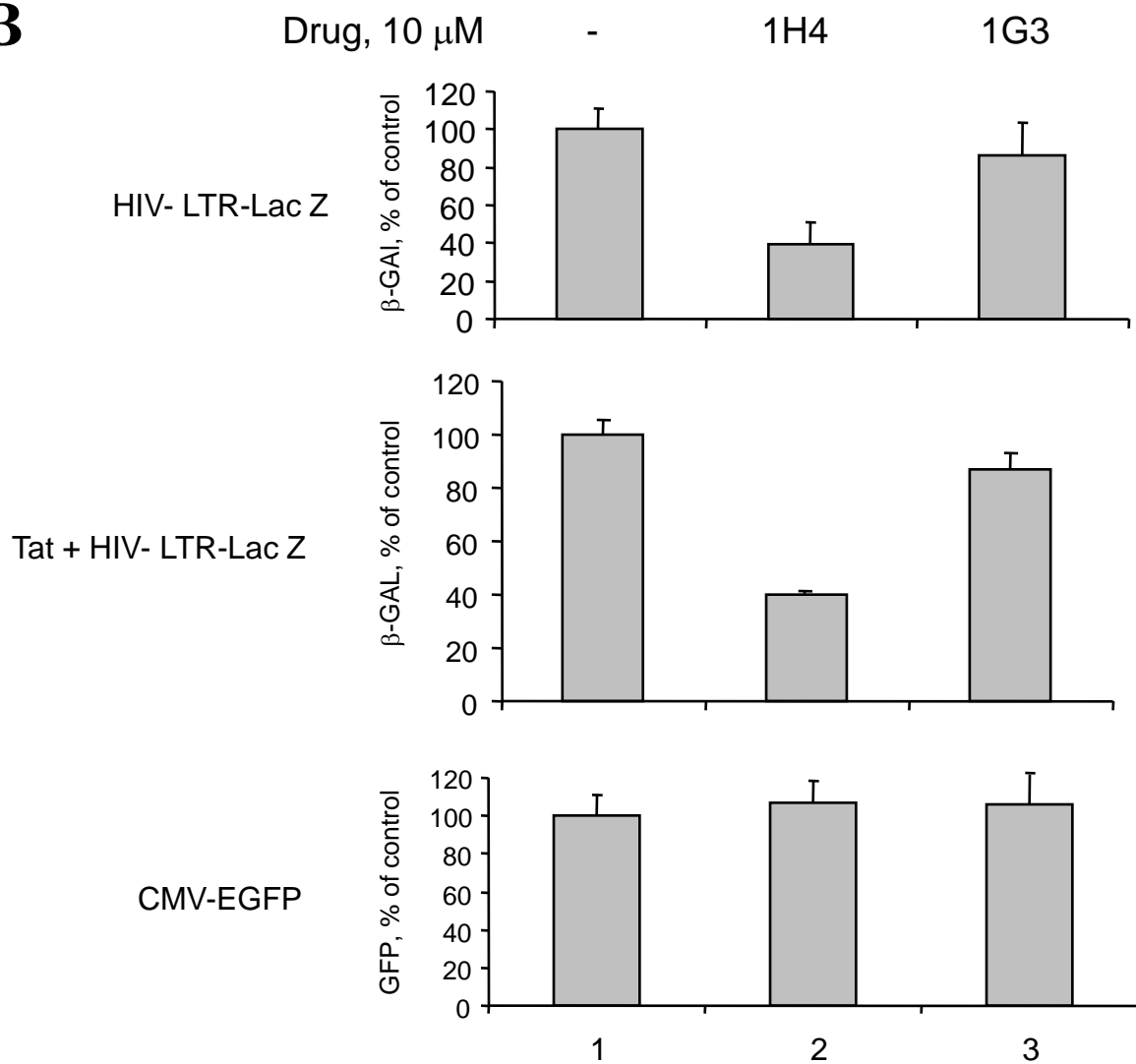

Supplement: Figure S2 — Effect of 1H4 on HIV-1 transcription, toxicity and CMV transcription. A. Inhibition of HIV-1 transcription and toxicity of PP1 inhibitors in CEM-GFP cells. CEM-GFP cells were infected with Adeno-Tat and then treated with the indicated concentrations of the PP1 inhibitors for 24 h. GFP fluorescence was measured in live cells. The cells were supplemented with propidium iodide (PI), and its fluorescence was measured. B. Effect of 1H4 on HIV-1 and CMV transcription. HEK293T cells were transfected with HIV-1 LTR-LacZ in the absence or presence of Tat expression vector (Tat). The cells were also co-transfected with CMV- EGFP-expression vector and treated with 10 µM 1H4 (lane 2) or 10 µM 1G3 (lane 3). Twenty-four hours after transfection, the cells were lysed and first analyzed on a luminescence spectrometer (LS50B, Perkin-Elmer) with an attached 96-well plate scanner at 480 nm excitation and at 510 nm emission for EGFP, and then analyzed for β-galactosidase activity using ONPG as a substrate. (PDF) [file pone.0039481.s002.pdf]
