# Supplementary material for: Small Molecules Targeted to a Non-Catalytic “RVxF” Binding Site of Protein Phosphatase-1 Inhibit HIV-1
Source: PLoS One. 2012 Jun 29;7(6):e39481. doi: 10.1371/journal.pone.0039481 (PMC3387161; doi:10.1371/journal.pone.0039481)
Supplement: Table S1 — Analysis of 262 small molecules for the inhibition of HIV-1 transcription in CEM GFP cells infected with Ad-Tat. Percent of inhibition is shown. Compounds chosen for further analysis are shown in gray. (PDF) [file pone.0039481.s003.pdf]

Table S1. Analysis of 262 small molecules for the inhibition of HIV-1 transcription in CEM GFP cells infected with Ad-Tat. Percent of inhibition is shown. Compounds chosen for further analysis are shown in gray.

Plate 1											
	1	2	3	4	5	6	7	8	9	10	11
A	92	8	1	0	78	27	65	32	80	30	23
B	46	24	42	42	69	74	99	53	34	25	73
C	95	54	55	59	56	52	98	41	61	32	53
D	57	82	57	56	54	71	46	49	39	14	55
E	46	40	30	60	54	49	74	41	39	64	35
F	42	52	50	56	52	51	60	63	46	36	94
G	33	45	80	50	46	41	46	0	46	94	32
H	53	86	51	96	33	72	94	36	56	69	52

Plate 2											
	1	2	3	4	5	6	7	8	9	10	11
A	60	2	-1	59	48	80	13	59	28	26	5
B	31	68	34	67	84	69	95	84	81	65	60
C	63	100	92	99	91	100	86	69	77	80	56
D	66	80	82	94	82	68	69	96	64	61	60
E	61	83	76	77	69	69	76	72	95	69	55
F	66	74	91	69	77	71	83	66	71	80	61
G	91	64	100	93	70	95	74	73	78	100	74
H	69	76	76	69	100	55	69	74	83	65	54

Plate 3											
	1	2	3	4	5	6	7	8	9	10	11
A	24	83	80	83	42	91	39	94	78		
B	23	57	56	53	63	45	70	74	93		
C	84	96	51	78	75	42	61	94	64		
D	59	62	53	75	48	40	87	57	54		
E	97	76	74	57	52	48	71	42	51		
F	76	53	76	66	62	62	56	95	48		
G	98	80	51	52	59	46	45	47			
H	48	44	43	72	79	71	90	90			
